# Editorial

**Published:** 1840-02-08

**Authors:** 


THE MEDICAL EXAMINER.

PHILADELPHIA, FEBRUARY 8, 1840.

We call the attention of our readers to some remarks on the treatment of jaundice, by Dr. James Jackson, for many years Professor of the Practice of Medicine at Harvard University. His long experience, and habits of close observation, give them unusual value. We have had some cases of the kind, and we have often derived great benefit from cupping over the liver, which we have always directed when the jaundice was attended with pain, or even decided uneasiness in the region of the liver. From the observations of Dr. Jackson, we find that much benefit follows even in cases where no decided pain is felt. Does not this prove that jaundice, if not decidedly inflammatory in many cases at the commencement, becomes so soon afterwards?

---

There is no better proof of the favour with which the Examiner has been received by the profession, than the unusually large proportion of elaborate and important memoirs which are now at our disposal. To our brethren who have aided us in our arduous enterprise, we feel most grateful,—and shall trust to their continued co-operation as a means of extending the usefulness of our work. The success of journals undertaken by members of the profession, has not hitherto been sufficient to meet the heavy expense of the enterprise: that the Examiner has formed an exception, is at once an evidence that our labours have been well received, and that the profession, as a body, are disposed to further the only practicable plan for sustaining a journal belonging to the profession,—we mean early and regular payments.

The more decidedly elaborate character of many of the articles of the Examiner will not prevent us from keeping up the usual variety of matter, and furnishing at as early a period as possible the important facts added to the science both in America and in Europe.

---

In the Eclectic Journal of Medicine, we find the following remark relative to the Medical Examiner:—"It contains short (somewhat too short) bibliographical notices."

The paging and printing of the Examiner have been adopted with a view to economy in the expenses of the journal, and in the expenses of postage of our subscribers. Hence, a person not accustomed to estimate the quantity of matter contained in a page, is not aware of the great length of many of the articles published in the Examiner, if opened out in the ordinary way. Two pages of it are just equal to three of the small type,—that is, of the journal matter of the Eclectic Journal—of course, to much more of the library department. For instance, we find that the bibliographical notice of Dr. Gross' work is decidedly longer in the Examiner than in the Eclectic Journal, (the standard which we suppose was in the mind of the editor.) Now, we would distinctly state that we do not estimate our articles by their length; but, at the same time, we are not willing that our articles should be looked upon as diminutive, because they are printed in a close and condensed form. That even a practised eye may be easily deceived, is shown by the remark of our estimable friend, the editor of the Eclectic Journal.

